# Preoperative prognostic prediction for stage I lung adenocarcinomas: Impact of the computed tomography features associated with the new histological grading system

**DOI:** 10.3389/fonc.2023.1103269

**Published:** 2023-01-31

**Authors:** Min Liang, Wei Tang, Fengwei Tan, Hui Zeng, Changyuan Guo, Feiyue Feng, Ning Wu

**Affiliations:** ^1^ Department of Diagnostic Radiology, National Cancer Center/National Clinical Research Center for Cancer/Cancer Hospital, Chinese Academy of Medical Sciences and Peking Union Medical College, Beijing, China; ^2^ Department of Thoracic Surgery, National Cancer Center/National Clinical Research Center for Cancer/Cancer Hospital, Chinese Academy of Medical Sciences and Peking Union Medical College, Beijing, China; ^3^ Department of Immunology and National Key Laboratory of Medical Molecular Biology, Institute of Basic Medical Sciences, Chinese Academy of Medical Sciences and Peking Union Medical College, Beijing, China; ^4^ Department of Pathology, National Cancer Center/National Clinical Research Center for Cancer/Cancer Hospital, Chinese Academy of Medical Sciences and Peking Union Medical College, Beijing, China; ^5^ Department of Nuclear Medicine (PET-CT Center), National Cancer Center/National Clinical Research Center for Cancer/Cancer Hospital, Chinese Academy of Medical Sciences and Peking Union Medical College, Beijing, China; ^6^ Department of Diagnostic Radiology, National Cancer Center/National Clinical Research Center for Cancer/Hebei Cancer Hospital, Chinese Academy of Medical Sciences, Langfang, China

**Keywords:** invasive lung adenocarcinoma, IASLC grading system, clinical T stage, preoperative CT imaging, prognostic model

## Abstract

**Objectives:**

This study aimed to identify the computed tomography (CT) features associated with the new International Association for the Study of Lung Cancer (IASLC) three-tiered grading system to improve the preoperative prediction of disease-free survival of stage I lung adenocarcinoma patients.

**Methods:**

The study included 379 patients. Ordinal logistic regression analysis was used to identify the independent predictors of IASLC grades. The first multivariate Cox regression model (Model 1) was based on the significant factors from the univariate analysis. The second multivariate model (Model 2) excluded the histologic grade and based only on preoperative factors.

**Results:**

Larger consolidation tumor ratio (OR=2.15, *P*<.001), whole tumor size (OR=1.74, *P*=.002), and higher CT value (OR=3.77, *P*=.001) were independent predictors of higher IASLC grade. Sixty patients experienced recurrences after 70.4 months of follow-up. Model 1 consisted of age (HR:1.05, *P*=.003), clinical T stage (HR:2.32, *P*<.001), histologic grade (HR:4.31, *P*<.001), and burrs sign (HR:5.96, *P*<.001). Model 2 consisted of age (HR,1.04; *P*=.015), clinical T stage (HR:2.49, *P*<.001), consolidation tumor ratio (HR:2.49, *P*=.016), whole tumor size (HR:2.81, *P*=.022), and the burrs sign (HR:4.55, *P*=.002). Model 1 had the best prognostic predictive performance, followed by Model 2, clinical T stage, and histologic grade.

**Conclusion:**

CTR (cut-off values of <25% and ≥75%) and whole tumor size (cut-off value of 17 mm) could stratify patients into different prognosis and be used as preoperative surrogates for the IASLC grading system. Integrating these CT features with clinical T staging can improve the preoperative prognostic prediction for stage I lung adenocarcinoma patients.

## Introduction

With the global popularization of low-dose computed tomography screening and the advancement of multi-slice spiral CT technology, non-small cell lung cancers (NSCLCs) are frequently detected early, and this has significantly reduced lung cancer mortality. Surgical resection is the preferred treatment for patients with stage I NSCLCs. No treatment is recommended after the resection of stage IA NSCLCs, and adjuvant chemotherapy is not routine for stage IB NSCLCs (1, 2). However, complete resection is not always curative; the 5-year disease-free survival (DFS) rate is 84.3% for stage IA NSCLC and 65.8% for stage IB NSCLC (3). Identifying patients at high risk of recurrence is vital for individualized follow-up strategies. Furthermore, tumor prognosis before surgery guides preoperative adjuvant therapy and the formulation of personalized treatment strategies for some patients who are medically contraindicated for surgical resection or refuse to undergo surgery because of poor performance status, older age, and complications.

It is well-known that T staging could stratify patients into different prognostic groups. However, patients with the same T stage are histologically heterogeneous. Several attempts have been made to integrate histological subtypes and T stages to improve prognostic prediction for patients with stage I lung adenocarcinoma (LUAD) (4, 5). A new histological grading scheme was proposed by the Pathology Committee of the International Association for the Study of Lung Cancer (IASLC) in 2020 and recommended by the fifth edition of the World Health Organization (WHO) classification of thoracic tumors in 2021. Several validation studies have confirmed its prognostic value (6–8). Our study attempted to combine the new IASLC three-tier grades with the T stages to further advance prognostic prediction for stage I LUAD.

We aimed to establish a nomogram based on preoperative predictors to improve the predictive capacity before treatment. Evaluating the association between the IASLC grading system and traditional CT imaging features will help identify additional preoperative predictors that are useful for prognostic stratification. We hypothesized that these radiologic features could be used as preoperative surrogates for IASLC histologic grades, and the model established by integrating these features with other preoperative predictors, including the clinical T stage, will demonstrate predictive performance comparable to that of the model directly based on the IASLC grading system and preoperative predictors.

## Materials and methods

The independent ethics committee approved the protocols for data collection, and the requirement for informed consent was waived because the study was retrospective.

### Patients and follow-up

**Figure 1:**
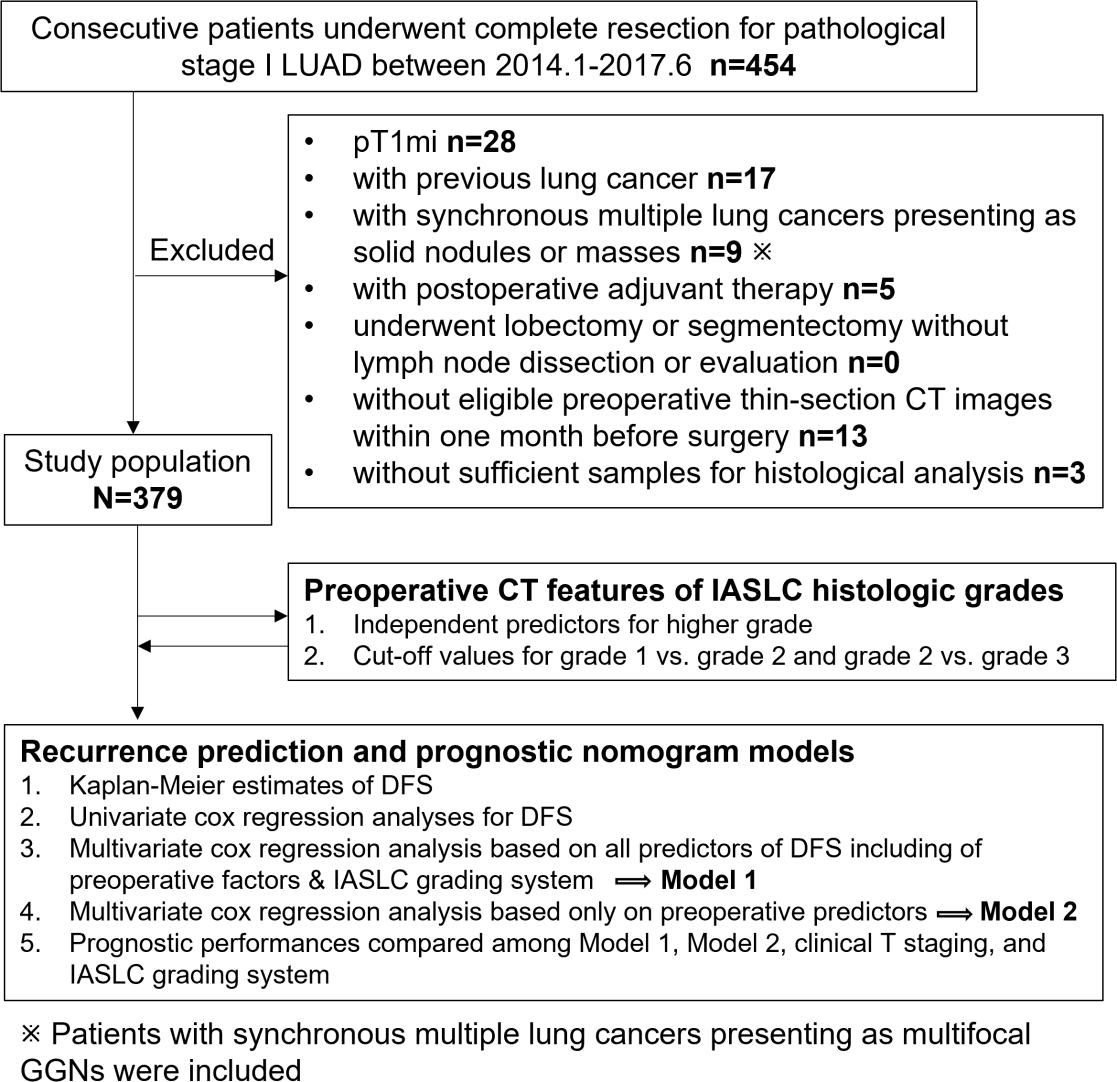
Flow chart of patient selection and study procedure. LUAD, lung adenocarcinoma; DFS, disease-free survival; GGN, ground glass nodule.

We reviewed the medical records of consecutive patients who underwent lobectomy or sublobar resection at our institution for primary pathological (p) stage I (T1N0M0 or T2aN0M0) LUAD between January 2014 and June 2017. In total, 454 cases were collected. All patients were staged using the eighth edition of the TNM Classification of Malignant Tumors (9). The exclusion criteria were as follows: (a) pT1mi cases (n=28); (b) patients with previous lung cancer (n=17); (c) patients who underwent lobectomy or segmentectomy without lymph node dissection or evaluation (n=0); (d) patients with synchronous multiple lung cancers presenting as solid nodules or masses (n=9); (e) patients who received postoperative adjuvant therapy (n=5); (f) patients without preoperative thin-section chest CT images within one month before surgery at our hospital or those with motion artifacts on CT images (n=13); and (g) patients with insufficient samples for histological analysis (n=3). Consequently, our study included 379 patients. To facilitate the understanding of our entire study, we showed the flowchart of patient selection and study procedure in **Figure 1**.

Follow-up for postoperative procedures began on the date of surgery. The endpoint of this study was any type of recurrence, including regional and distant recurrence. DFS was defined as the duration from radical surgery for lung cancer to the first recurrence. The time of the last follow-up was used for the censored patients. All recurrences were confirmed by clinical, radiologic, and pathologic assessments and classified as regional or distant. During the postoperative follow-up, histologic profiles were reviewed for cases of new lung nodules or masses suspected to be metachronous primary tumors to ascertain or rule out recurrence.

### CT Image acquisition

Chest images were obtained using 64-detector row CT scanners (LightSpeed VCT, Discovery CT750 HD, Optima CT660, GE Healthcare, Milwaukee, WI, USA; and Toshiba Aquilion, Toshiba Medical Systems, Japan) set at tube voltage of 120 kV, tube currents of 270 mA or auto-controlled current, 512 × 512-pixel resolution, rotation time of 0.5 s, and pitch of 0.984 or 0.992. The reconstructed section thickness was 1 or 1.25 mm, with an interval of 0.8 mm. For enhanced CT scans, iopromide was administered at 80-90 ml (1.5 ml/kg body weight) at a rate of 2.5-3.0 ml/s with a power injector. CT scan was started 35 seconds after completion of the injection.

### Radiologic variables

All CT images were reviewed by two chest radiologists (M.L. and W.T., with 4 and 18 years of experience in chest CT, respectively) blinded to the histopathology results, and all decisions were reached by consensus. Thin-section CT images were analyzed with a lung window width of 1500–1600 Hounsfield units (HU) at the level of -600 to -650 HU and a mediastinal window width of 350–380 HU at the level of 40 to 60 HU. The following data were recorded: tumor location, CT appearances (including non-solid, part-solid, and solid), whole tumor size, solid portion size, clinical T (cT) stage, consolidation tumor ratio (CTR), mean CT value, distance from the tumor to the pleura, tumor shape (regular or irregular), lesion margins (clear or blurred), presence of the burrs sign, lobulation, vacuole, pleural adhesion, pleural retraction, thickening of the adjacent pleura, and presence or absence of multifocal ground glass nodules (GGNs). For multifocal GGNs, all data were based only on the lesion with the highest clinical T stage.

The whole tumor size was expressed as the mean diameter, which was the average of the tumor length and width. The length and width of the tumor were measured on axial or reformatted images, which showed the maximum tumor size. The cT category (cTis to cT2a) was determined according to the eighth edition of the staging system for lung cancer, which is based on the maximum diameter of the solid component for part-solid lesions and the maximum diameter of the whole tumor for solid lesions (10). The CTR was calculated as the division of the maximum diameter of the consolidation by the maximum tumor diameter and recorded as 0%, >0 and <25%, ≥25% and <50%, ≥50% and <75%, ≥75% and <100%, or 100%. The mean CT value was measured by manually outlining the contour of the entire tumor in the lung window. Blood vessels, small bronchi, and cysts were avoided as much as possible; however, some inescapable vacuoles, small blood vessels, and small bronchi were included. The distance between the tumor and pleura refers to the vertical length of the tumor edge to the nearest visceral pleura.

### Histopathologic evaluation

All enrolled pathological specimens were reviewed by a senior pathologist with >10 years of experience in diagnosing thoracic tumors, who was blinded to patient clinical outcomes. All cases were classified as grade 1, 2, or 3 according to the 2020 IASLC three-tier grading system (7).

### Construction and evaluation of the nomogram

Variables that were significant in the univariate analysis were further analyzed using multivariate analysis. Cox regression analyses were performed to identify the independent prognostic factors based on demographic and preoperative CT imaging features with or without IASLC histologic grade. Nomograms were used for DFS prediction visualization. Corresponding calibration curves were generated to evaluate the accuracy of the nomogram. The prognostic performances of the nomograms and other conventional predictors of DFS were compared using the time-dependent Harrell concordance index (C-index). Furthermore, decision curve analysis (DCA) was used to evaluate the clinical utility by measuring the net benefits at different threshold probabilities.

### Statistical analysis

The clinicopathological and imaging features were compared among histologic grade 1, 2, and 3 groups and between the recurrence and non-recurrence groups, respectively. For the quantitative variables, comparisons of the three groups were evaluated using one-way ANOVA or Kruskal-Wallis test analysis, and comparisons of two groups were performed using the T-test or Wilcoxon rank-sum test. The chi-squared test or Fisher’s exact test was used for intra- or inter-group comparisons of the categorical data.

Ordinal logistic regression analysis was performed to identify the independent risk factors for IASLC histological grading system. Continuous variables that were statistically significant based on the one-way ANOVA (or Kruskal-Wallis test) or chi-squared test (or Fisher’s exact test) were converted to categorical/ordered variables according to the cut-off values for grade 1 vs. grade 2 and grade 2 vs. grade 3 determined by receiver operating characteristic curves.

The Kaplan–Meier method was used to generate DFS curves, and differences in survival curves were compared using the log-rank test. Univariate and multivariate Cox regression analyses were performed to identify the independent prognostic factors for DFS. These independent predictors were used to establish nomograms using the “rms” package. Calibrations of nomograms were used for internal validation by the 1000 bootstrap resampling procedure. Calibration curves were generated by plotting the predicted and actual DFS to establish the accuracy of the nomograms. The time-dependent C-index was plotted using the “pec” package. The clinical utility of the nomogram was assessed using the DCA and the “rmda” package.

SPSS version 24.0 (IBM, Armonk, NY, USA) and R for Windows version 4.12 (http://www.r-project.org/) were used for the statistical analyses. Statistical significance was set at *P*<0.05.

## Results

### Demographic, clinicopathologic, and radiological characteristics and outcomes of patients

Based on the IASLC grading system, 379 patients with LUADs were classified as follows: grade 1 (n=57, 15%), grade 2 (n=233, 61.5%), or grade 3 (n=89, 23.5%). Higher grades were associated with male sex, former or current smoking, and more pack years. Furthermore, higher-grade tumors showed aggressive features reflected in the CTR, whole tumor size, stage, lymph vascular invasion, and visceral pleural invasion. In addition, higher tumor grades were associated with higher frequencies of lobectomy and lymph node dissection. All *P*-values above were less than 0.05 (**Table 1**).

**Table 1:** Patients’ and nodules’ characteristics based on histologic grades and outcomes in p-stage I adenocarcinoma.

Variable	Total (n=379)	Histologic Grade				Recurrence		
		Grade 1 (n=57)	Grade 2 (n=233)	Grade 3 (n=89)	P	NO (n=319)	Yes (n=60)	P
Sex					0.014			0.003
Female	229 (60.4)	37 (64.9)	150 (64.4)	42 (47.2)		203 (63.3)	26 (43.3)	
Male	150 (39.6)	20 (35.1)	83 (35.6)	47 (52.8)		116 (36.4)	34 (56.7)	
Median age (IQR), y	59 (11)	58 (9)	59 (12)	60 (13)	0.949	58 (11)	61 (9)	0.078
Smoking					<0.001			0.004
Never	273 (72)	49 (86.0)	177 (76.0)	47 (52.8)		240 (75.2)	33 (55.0)	
Former	54 (14.2)	4 (7.0)	32 (13.7)	18 (20.2)		42 (13.2)	12 (20.0)	
Current	52 (13.7)	4 (7.0)	24 (10.3)	24 (27.0)		37 (11.6)	15 (25.0)	
Pack-years	29.3 (20.5)	30.5 (24.6)	24.6 (13.8)	35.2 (25.3)	0.039	25 (25)	30 (31.3)	0.069
History of other cancer	39 (10.3)	3 (5.3)	28 (12.0)	8 (9.0)	0.488	35 (11.0)	4 (6.7)	0.258
Family history of lung cancer	52 (13.7)	6 (10.5)	35 (15.0)	11 (12.4)	0.618	43 (13.5)	9 (15.0)	0.754
Multifocal GGNs	119 (31.5)	22 (38.6)	71 (30.6)	26 (29.2)	0.442	99 (32.1)	20 (33.3)	0.736
Nodule location					0.024			0.799
RUL	149 (39.3)	23 (40.4)	90 (38.6)	36 (40.4)		125 (39.2)	24 (40)	
RML	22 (5.8)	0 (0)	14 (6.0)	8 (9.0)		17 (5.3)	5 (8.3)	
RLL	67 (17.7)	7 (12.3)	43 (18.5)	17 (19.1)		55 (17.2)	12 (20.0)	
LUL	100 (26.4)	24 (42.1)	58 (24.9)	18 (20.2)		87 (27.3)	13 (21.7)	
LLL	41 (10.8)	3 (5.3)	28 (12.0)	10 (11.2)		35 (11.0)	6 (10.0)	
CT findings					<0.001			<0.001
Pure GGN (CTR=0)	79 (20.8)	42 (73.7)	37 (15.9)	0 (0)		79 (24.8)	0 (0)	
Part-solid nodule	197 (52.0)	15 (26.3)	157 (67.4)	25 (28.1)	<0.001	177 (55.5)	20 (33.3)	<0.001
CTR<0.25	86 (22.7)	12 (21.1)	72 (30.9)	2 (2.2)		86 (27.0)	0 (0)	
0.25≤CTR<0.5	41 (10.8)	1 (1.8)	36 (15.5)	4 (4.5)		38 (11.9)	3 (5.0)	
0.5≤CTR<0.75	29 (7.7)	2 (3.5)	22 (9.4)	5 (5.6)		24 (7.5)	5 (8.3)	
CTR≥0.75	41 (10.8)	0 (0)	27 (11.6)	14 (15.7)		29 (9.1)	12 (20)	
Solid nodule (CTR=1)	103 (27.2)	0 (0)	39 (16.7)	64 (71.9)		63 (19.7)	40 (66.7)	
Whole tumor size (IQR), mm	17.4 (8.5)	14.9 (10.6)	16.7 (7.7)	19.5 (8.2)	<0.001	16.4 (7.2)	22.4 (9.0)	<0.001
Clinical T stage					<0.001			<0.001
cTis	79 (20.8)	43 (75.4)	36 (15.5)	0 (0)		79 (24.8)	0 (0)	
cT1mi	40 (10.6)	4 (7.0)	36 (15.5)	0 (0)		40 (12.5)	0 (0)	
cT1a	64 (16.9)	8 (14.0)	54 (23.2)	2 (2.2)		64 (20.1)	0 (0)	
cT1b	123 (32.5)	2 (3.5)	82 (35.2)	39 (43.8)		105 (32.9)	18 (30.0)	
cT1c	63 (16.6)	0 (0)	22 (9.4)	41 (46.1)		29 (9.1)	34 (56.7)	
cT2a	10 (2.6)	0 (0)	3 (1.3)	7 (7.9)		2 (0.6)	8 (13.3)	
Pleural retraction	185 (48.8)	11 (19.3)	126 (54.1)	48 (53.9)	<0.001	156 (48.9)	29 (48.3)	0.146
Pleural adhesion	94 (24.8)	17 (29.8)	54 (23.2)	23 (25.8)	<0.001	74 (23.2)	20 (33.3)	0.146
Thickening of pleura	121 (31.9)	12 (21.1)	76 (32.6)	33 (37.1)	0.120	97 (30.4)	24 (40.0)	0.144
Surgical mode					0.012			0.286
Sublobar resection	69 (18.2)	14 (24.6)	48 (20.6)	7 (7.9)		61 (19.1)	8 (13.3)	
Lobar resection	310 (81.8)	43 (75.4)	185 (79.4)	82 (92.1)		258 (80.9)	52 (86.7)	
Lymph node dissection					0.024			0.195
Yes	360 (95.0)	50 (87.7)	224 (96.1)	86 (96.6)		301 (94.4)	59 (98.3)	
No†	19 (5.0)	7 (12.3)	9 (3.9)	3 (3.4)		18 (5.6)	1 (1.7)	
Pathologic T stage††					<0.001			<0.001
pT1a	50 (13.2)	20 (35.1)	29 (12.4)	1 (1.1)		50 (15.7)	0 (0)	
pT1b	198 (52.2)	27 (47.4)	130 (55.8)	41 (46.1)		179 (56.1)	19 (31.7)	
pT1c	107 (28.2)	10 (17.5)	60 (25.8)	37 (41.6)		78 (24.5)	29 (48.3)	
pT2a	24 (6.3)	0 (0)	14 (6)	10 (11.2)		12 (3.8)	12 (20)	
Visceral pleura invasion†††	21 (5.5)	0 (0)	12 (5.2)	9 (10.1)	0.031	11 (3.4)	10 (16.7)	<0.001
Lymph vascular invasion	18 (4.7)	0 (0)	6 (2.6)	12 (13.5)	<0.001	6 (1.9)	12 (20.0)	<0.001
Median follow-up time (IQR), month	70.4 (60.2-79.0)	75.0 (66.9-83.5)	70.0 (60.2-78.7)	64.0 (49.0-76.8)	0.004			
Recurrence	60 (15.9%)	0 (0)	15 (6.5)	45 (51.1)	<0.001			

IQR, interquartile range; CTR, consolidation tumor ratio; PSN, part-solid nodule; GGN, ground glass nodule; RUL, right upper lobe; RML, right middle lobe; RLL, right lower lobe; LUL, left upper lobe; LLL, left lower lobe; GG/L, ground glass/lepidic.

Pack-years, number of packs smoked per day × number of years smoked.

Qualitative data are expressed as n (%), and quantitative data as median (IQR).

Values are numbers of patients (percentages) unless otherwise indicated.

The median follow-up times were calculated using the Kaplan-Meier method, and P value were calculated by the log-rank test.

The other P values were calculated by One-way ANOVA (or Kruskal-Wallis test) and T-test (or Wilcoxon rank-sum test) for continuous variables, and Chi-square test or the Fisher’s exact test for categorical variables.

†19 patients who did not undergo lymph node dissection or evaluation underwent wedge resection.

††The pathologic T staging was determined according to the 8th edition of TNM classification.

†††All were confirmed by elastic fiber staining, 16 cases with a maximum invasion diameter of less than 3 cm pathologically, upstage to pT2a due to visceral pleura invasion

After 70.4 (interquartile range [IQR]: 60.2–79.0) months, 60 (15.9%) patients had recurrence. The cases of recurrence were distributed as follows: 0, grade 1 group; 15 (6.5%), grade 2 group; and grade 3 group, 45 (51.1%). In addition to the higher grade, recurrence was also associated with male sex, former or current smoking, higher CTR and whole tumor size, higher clinical and pathological T stage, and the presence of visceral pleural invasion and lymph vascular invasion. All *P*-values above were less than 0.05 (**Table 1**).

### Association between CT features and IASLC histologic grades

Exploring the association between CT features and histologic grades could not only identify CT features that have predictive value for IASLC grading system, but also convert these features into categorical variables according to various grade cut-offs. After such transformation, the preoperative CT features were closely associated with histologic grades and could be considered as preoperative surrogates for IASLC grading system.

There were significant differences among three grades in following CT appearances: tumor location, CTR, whole tumor size, mean CT value, the presences of burrs sign, lobulated sign, blurred margin, and pleural adhesion or retraction. The cut-off values for grade 1 vs. grade 2 and grade 2 vs. grade 3 were -420 HU (area under the curve [AUC], 95% CI: 0.921 [0.891-0.950], *P*<.001) and -205 HU (AUC [95% CI]: 0.879 [0.840-0.918], *P<*.001) for the mean CT value, 12 mm (AUC [95% CI]: 0.633 [0.548-0.718]; *P=*.001) and 17 mm (AUC [95% CI]: 0.673 [0.612-0.734]; *P<*.001) for the whole tumor size, and >0 to <25% (AUC [95% CI]: 0.884 [0.846-0.923]; *P<*.001) and ≥75% to <100% (AUC [95%CI]: 0.878 [0.843-0.913]; *P<*.001) for CTR, respectively. The whole tumor size and mean CT values were converted to categorical variables based on the cut-off values.

**Figure 2:**
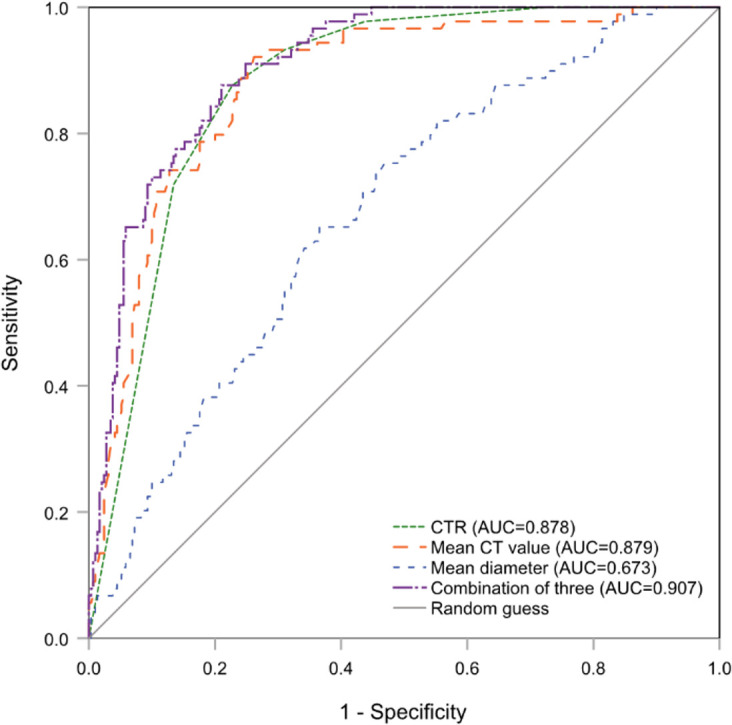
The ROC curves of CTR, mean CT value and whole tumor size (mean diameter) alone and combination for predicting histologic grade 3. ROC, receiver operating characteristic; CTR, consolidation tumor ratio.

The Brant test suggested that the parallel line assumption held (χ2 = 20.52, *P*=0.153). The ordinal regression analysis results showed that only a higher CTR (OR=2.15, 95% CI:1.53-3.00, *P<*.001), larger whole tumor size (OR=1.74, 95% CI:1.23-2.48, *P=*.002), and higher CT value (OR=3.77, 95% CI:1.77-8.02, *P=*.001) were independent risk factors for higher histologic grade (**Table 2**). The AUC value for conjoining the above three independent factors to predict grade 3 was 0.907 (95% CI:0.877-0,937; *P<*.001), and it was not significantly different from the AUC for using the mean CT value or CTR alone (**Figure 2**).

**Table 2:** Differences among the three histologic grades and results of multivariate ordered logistic regression analysis.

Variable	Parametric and nonparametric test ^a^		Multivariate ordered logistic regression analysis ^d^	
	Statistic	P	OR (95%CI)	P
Female (vs. male)	8.52	0.014	1.04 (0.55-1.95)	0.914
Smoking	24.88	<0.001		
Never			0.57 (0.25-1.32)	0.189
Current			1.10 (0.44-2.75)	0.832
Former			1	–
Tumor location	17.70	0.024		
RUL			1	–
RML			1.12 (0.37-3.35)	0.839
RLL			1.04 (0.52-2.06)	0.921
LUL			0.78 (0.43-1.43)	0.423
LLL			1.33 (0.58-3.07)	0.502
CTR^b^	238.34	<0.001	2.15 (1.53-3.00)	<0.001
Whole tumor size (mm)	16.12	<0.001	1.74 (1.23-2.48) ^c^	0.002
Mean CT value (HU)	172.31	<0.001	3.77 (1.77-8.02) ^c^	0.001
Irregular shape (vs. regular)	1.48	0.477	–	–
Burrs sign (vs. absent)	95.37	<0.001	0.82 (0.38-1.76)	0.612
Lobulated sign (vs. absent)	19.63	<0.001	1.40 (0.82-2.39)	0.217
Vacuole sign (vs. absent)	0.20	0.907	–	–
Blurred margin (vs. clear)	9.89	0.007	0.74 (0.44-1.26)	0.264
Relationship between tumor and pleura	28.14	<0.001		
Pleural adhesion			1	–
Pleural retraction			1.26 (0.64-2.49)	0.511
Neither of the above			1.55 (0.84-2.85)	0.159
Distance of tumor to pleura (mm)	0.05	0.955	–	–
Pleura thickening (vs. absent)	3.96	0.138	–	–

OR, odds ratio; CI, confidence interval; CTR, consolidation tumor ratio.

a Parametric test used One-way ANOVA or Kruskal-Wallis test, and non-parametric tests used Chi square test or the Fisher’s exact test.

b CTR with an increment of 25% for part-solid tumors, CTR=0 for pure GGN, and CTR=100% for solid tumors.

c Whole tumor size and mean CT value were converted into categorical variables by cut-offs from the corresponding ROC curves for multivariate ordinal logistic regression analysis.

d The multivariable ordered regression model expressed in odd ratios (ORs) and has a goodness-of-fit Index of 0.916.

### Kaplan-Meier estimates of DFS

**Figure 3:**
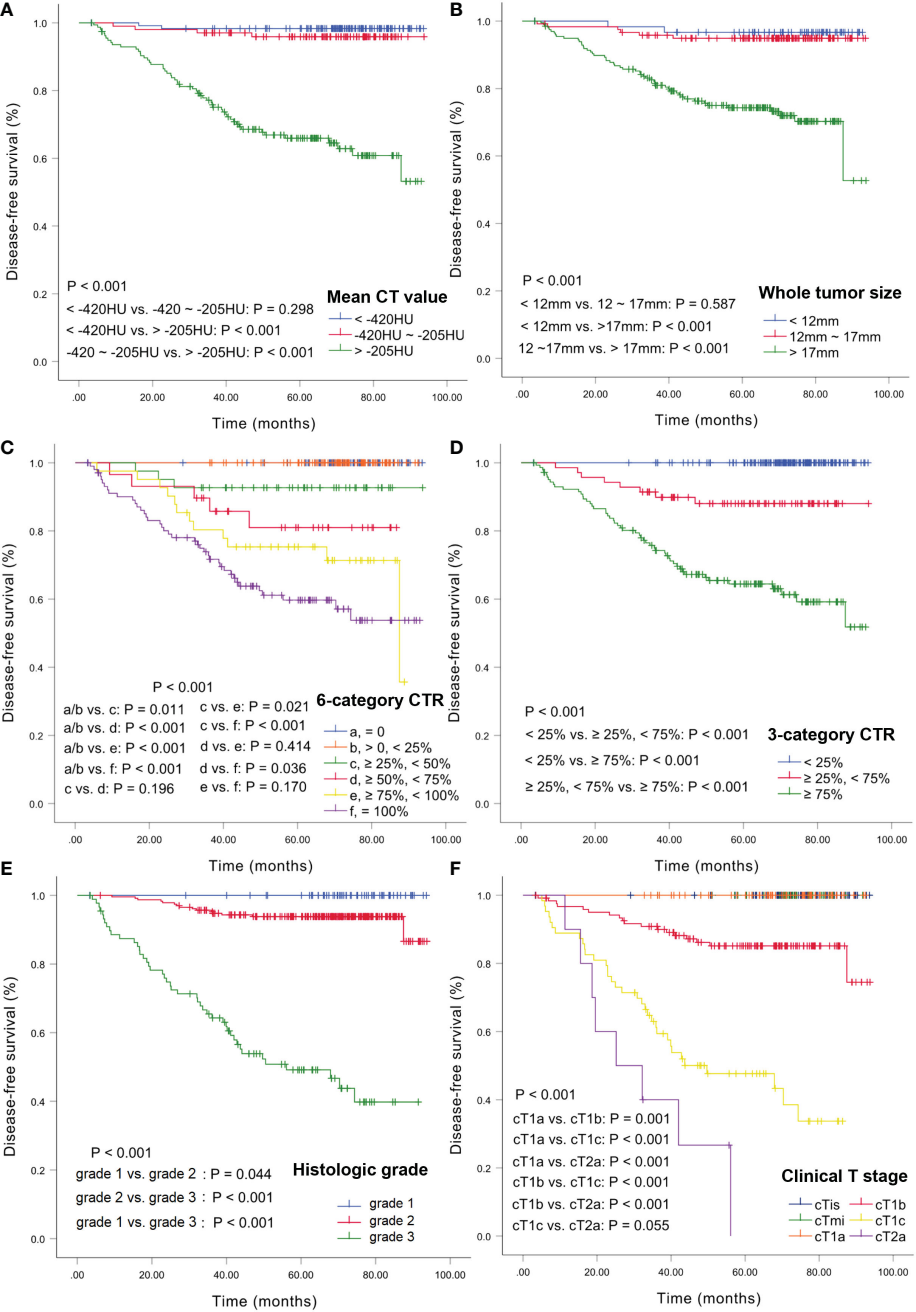
Kaplan-Meier estimates of DFS. DFS curves according to CT values **(A)**, whole tumor size **(B)**, 6-category CTR **(C)**, 3-category CTR **(D)**, histologic grade **(E)**, and clinical T stage **(F)**. DFS, disease-free survival; CTR, consolidation tumor ratio.

DFS curves were plotted using the Kaplan-Meier method based on the mean CT value, whole tumor size, 6- and 3-category CTR, cT stage, and histologic grade (**Figure 3**). The 3- and 5-year DFS rates for the CT values were 98.3% and 98.3% for <-420 HU; 97.0% and 95.9% for -420 HU to -205 HU, and 76.5% and 65.9% for >-205 HU, respectively. There was no significant difference between the rates for <-420 HU and -420 to -205 HU (*P=*0.298) (**Figure 3A**). The 3-year and 5-year DFS rates for the tumor sizes were 98.3% and 96.7% for <12 mm, 95.8% and 94.9% for 12–17 mm, and 82.0% and 74.2% for >17 mm, respectively, and there was no significant difference between the rates for <12 mm and 12–17 mm (*P=*0.587) (**Figure 3B**). For CTR, the 3-year and 5-year DFS rates were 100% and 100% for CTR=0 and 0<CTR<25%, 92.7% and 92.7% for 25%≤CTR<50%, 89.7% and 81.0% for 50%≤CTR<75%, 80.3% and 75.3% for 75%≤CTR<100%, and 72.5% and 59.3% for CTR=100%, respectively, and there were no statistical differences between any two adjacent curves except for CTR<25% and 25%≤CTR<50% (**Figure 3C**). The 3-year and 5-year DFS rates were 100% and 100% for CTR<25%, 91.4% and 88% for 25%≤CTR<75%, 74.8% and 64.2% for CTR≥75%, and there were significant differences among these three survival curves (all *P<*.001) (**Figure 3D**). For the histologic grades, the 3- and 5-year DFS rates were 100% and 100% for grade 1, 95.2% and 93.8% for grade 2, and 63.9% and 48.5% for grade 3, respectively, and there were significant differences among these curves (**Figure 3E**). The 3- and 5-year DFS rates were 100% and 100% for cTis/cT1mi/cT1a, 90.8% and 85.1% for cT1b, 60.5% and 46.7% for cT1c, and 40% and 0% for cT2a, respectively, and the log-rank test revealed significant differences among these curves (all *P<*.001) (**Figure 3F**).

### Cox regression analyses for DFS

Based on the results of Kaplan-Meier methods, we converted the CT value and whole tumor size into binary variables before Cox regression analysis: CT values: ≤-205 HU and >-205 HU; whole tumor size: ≤17 mm and >17 mm. CTR was transformed into an ordered 3-category variable with the following ranges: <25%, ≥25% to <75%, and ≥ 75%. Considering that cTis, cTmi, and cT1a were not associated with the risk of recurrence, they were grouped into one category. Univariate Cox analysis showed that sex, age, current or former smoking status, histologic grade, cT stage, whole tumor size, CTR, CT value, blurred margin, presence of burrs sign, and lobulation were associated with DFS. Multivariate Cox regression analysis showed that age (HR:1.05, 95% CI: 1.02-1.09, *P=*.003), burrs sign (HR:5.96, 95% CI:2.30-15.43, P<.001), cT stage (HR:2.32, 95% CI:1.53-3.52, *P<*.001), and histologic grade (HR:4.31, 95% CI:2.28-8.14, *P<*.001) were independent predictors of DFS. We conducted multivariate Cox regression analysis once again after excluding the histologic grade and based only on sex, age, smoking history, and preoperative imaging factors. Age (HR: 1.04, 95% CI: 1.01-1.08, *P*=0.015), burrs sign (HR: 4.55, 95% CI: 1.73-11.95, *P=*.002), cT stage (HR: 2.49, 95% CI: 1.58-3.93, *P<*.001), whole tumor size >17 mm (HR: 2.81, 95% CI: 1.16-6.77, *P=*.022), and CTR (HR: 2.49, 95% CI:1.19-5.25, *P*=.016) were considered independent prognostic factors for DFS (**Table 3**).

**Table 3:** Univariate and multivariate cox regression analysis for disease-free survival.

Variable	Univariate		Multivariate			
	HR (95% CI)	P	with histologic grade		without histologic grade	
			HR (95%CI)	P	HR (95%CI)	P
Female (vs. male)	0.47 (0.28-0.78)	0.003	0.67 (0.34-1.31)	0.240	0.72 (0.42-1.22)	0.219
Age (per 1-year increase)	1.03 (1.01-1.07)	0.046	1.05 (1.02-1.09)	0.003	1.04 (1.01-1.08)	0.015
Smoking (vs. never)		0.004		0.643		0.806
Current	2.70 (1.46-4.97)	0.001	0.69 (0.31-1.55)	0.369	1.21 (0.55-2.65)	0.631
Former	1.93 (0.10-3.74)	0.051	0.77 (0.34-1.75)	0.530	0.95 (0.42-2.13)	0.897
Pack-years	1.01 (1.00-1.03)	0.056	–	–	–	–
History of other cancer	0.17 (0.02-1.50)	0.110	–	–	–	–
Family history of lung cancer	1.14 (0.56-2.32)	0.712	–	–	–	–
Multifocal GGNs	1.11 (0.65-1.89)	0.713	–	–	–	–
Nodule location (vs. RUL)		0.820	–	–	–	–
RML	1.34 (0.51-3.52)	0.552	–	–	–	–
RLL	1.08 (0.54-2.16)	0.824	–	–	–	–
LUL	0.76 (0.39-1.50)	0.428	–	–	–	–
LLL	0.86 (0.35-2.10)	0.735	–	–	–	–
Clinical T stage**^#^**	4.34 (3.20-5.90)	< 0.001	2.32 (1.53-3.52)	< 0.001	2.49 (1.58-3.93)	< 0.001
CTR*	2.29 (1.80-2.90)	< 0.001	1.54 (0.70-3.38)	0.280	2.49 (1.19-5.25)	0.016
Whole tumor size (vs. ≤17mm)	1.14 (1.10-1.18)	< 0.001	2.18 (0.92-5.16)	0.076	2.81 (1.16-6.77)	0.022
Mean CT value (vs. ≤-205HU)	1.01 (1.01-1.01)	< 0.001	0.42 (0.09-1.91)	0.261	0.53 (0.13-2.14)	0.368
Irregular shape (vs. regular)	1.17 (0.69-1.99)	0.566	–	–	–	–
Burrs sign (vs. absent)	19.43 (7.76-48.65)	< 0.001	5.96 (2.30-15.43)	< 0.001	4.55 (1.73-11.95)	0.002
Lobulated sign (vs. absent)	2.29 (1.29-4.07)	0.005	0.83 (0.42-1.64)	0.587	0.82 (0.42-1.61)	0.564
Vacuole sign (vs. absent)	1.07 (0.57-2.01)	0.838	–	–	–	–
Blurred margin (vs. clear)	2.45 (1.44-4.18)	0.001	0.77 (0.42-1.42)	0.406	0.74 (0.41-1.31)	0.298
Relationship between tumor and pleura (vs. neither)		0.224	–	–	–	–
Pleural adhesion	1.93 (0.92-4.06)	0.084	–	–	–	–
Pleural retraction	1.53 (0.77-3.07)	0.229	–	–	–	–
Distance of tumor to pleura (mm)	1.01 (0.98-1.05)	0.417	–	–	–	–
Thickening of adjacent pleura	1.43 (0.85-2.41)	0.182	–	–	–	–
Histologic grade**^**	12.16 (6.85-21.57)	< 0.001	4.31 (2.28-8.14)	< 0.001	–	–

HR, hazard ratio; CI, confidence interval; CTR, consolidation/tumor ratio; GGNs, ground glass nodules.

Pack-years, number of packs smoked per day × number of years smoked.

**^#^** cT stage: cTis/cT1mi/cT1a, cT1b, cT1c, cT2a.

*CTR: <25%, ≥25% to <75%, ≥75%.

**^**Histologic grade: grade 1, grade 2, grade 3.

### Nomogram and model performances

**Figure 4:**
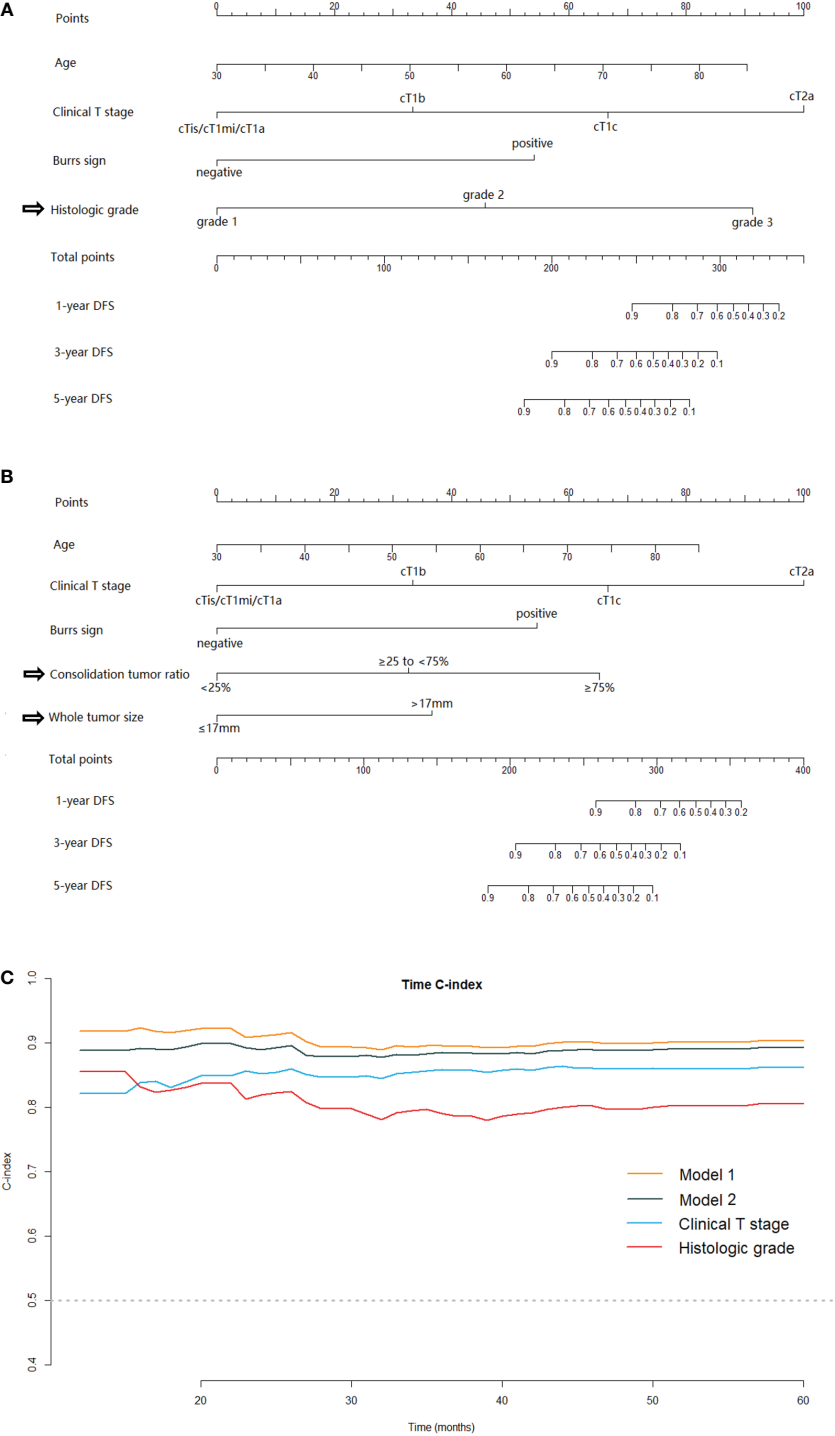
The nomogram models for predicting DFS for patients with pathological stage I LUAD and the C-index estimates over time. Model-1 **(A)** and Model-2 **(B)** for predicting the 1-, 3‐ and 5‐year DFS rates. **(C) **The time‐dependent C‐index of model 1, model 2, clinical T stage and histologic grade. DFS, disease-free survival; LUAD, lung adenocarcinoma.

**Figure 5:**
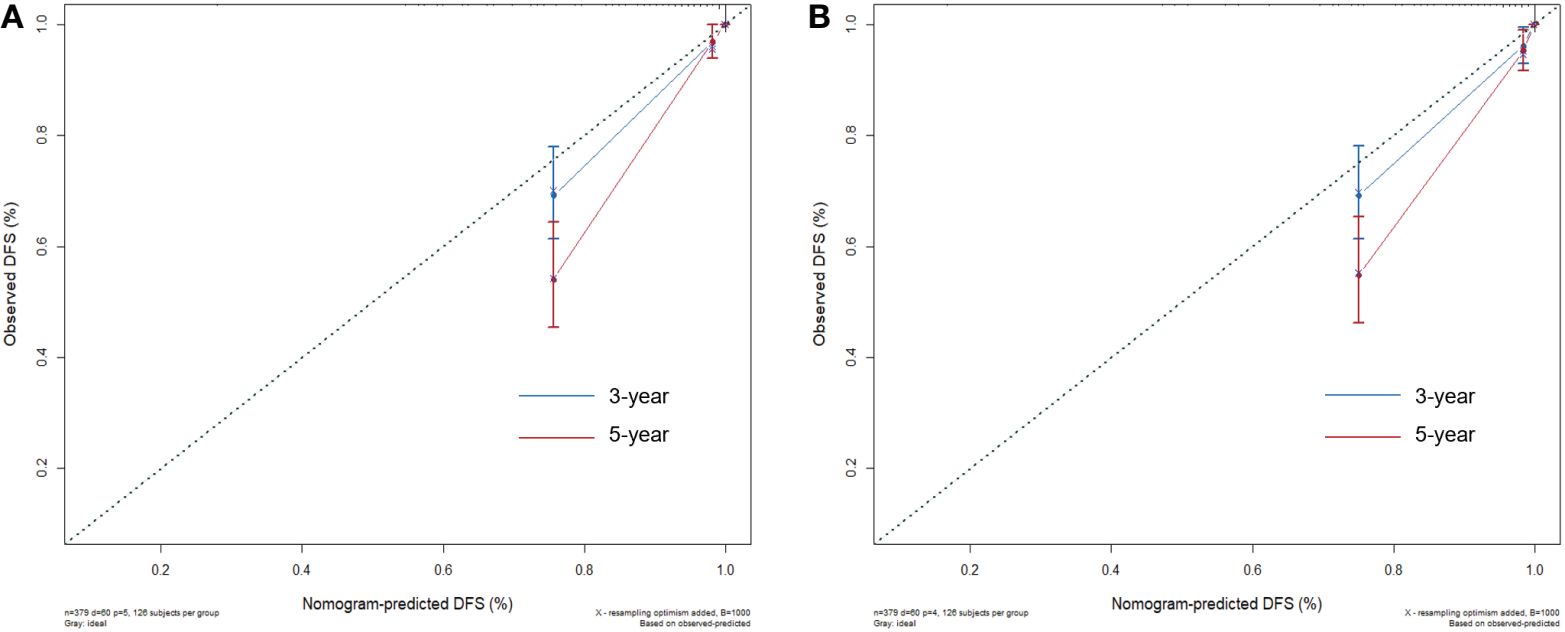
The calibration plots for evaluating the nomogram models. The calibration curves of Model 1 **(A)** and Model 2 **(B)** for predicting the 3‐ and 5‐year DFS.

**Figure 6:**
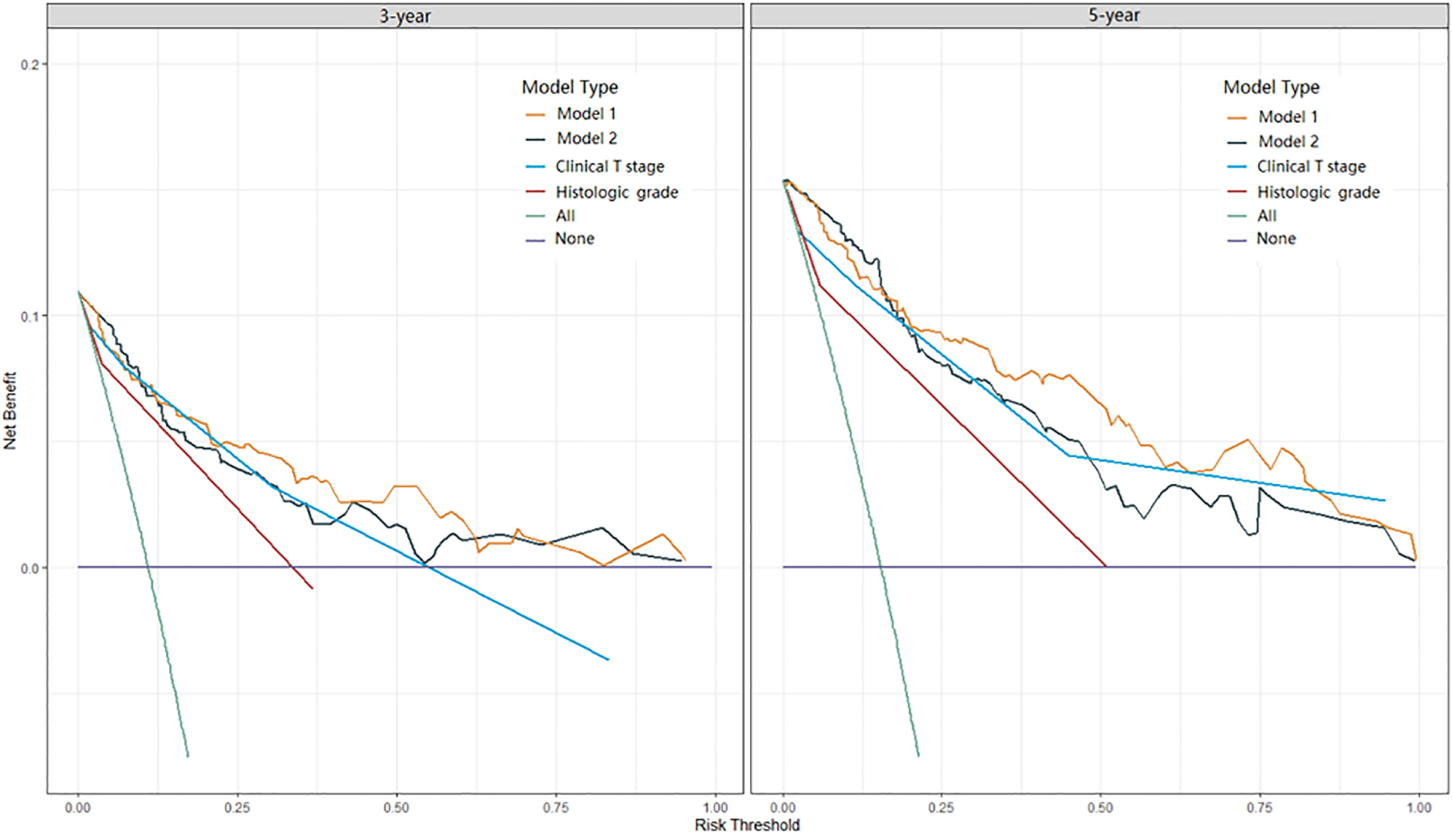
DCA curves of Model 1, Model 2, clinical T stage, and histologic grade in predicting 3-year and 5-year DFS. DCA, decision curve analysis.

Two nomograms for DFS were established by integrating independent prognostic indicators in the two multivariate Cox analyses. Model 1 consisted of age, histologic grade, cT stage, and burrs sign (**Figure 4A**). The predictors for Model 2 were identical to those for Model 1, except that the histologic grade was replaced with CTR and whole tumor size (**Figure 4B**). The approximate 1-, 3-, and 5-year DFS rates were calculated according to these models. The time-dependent C-index showed that the prognostic capacity of Model 1 was slightly superior to that of Model 2, followed by the cT stage and histologic grade, and the prognostic performances of the two models were stable over time (**Figure 4C**). The calibration plots revealed that both nomogram models showed satisfactory agreement between the prediction outcome and actual observation for the 3- and 5‐year DFS (**Figure 5**). The DCA curves showed that the clinical utility of Model 1 was slightly higher than that of Model 2, which was similar to the utility of cT staging alone (**Figure 6**).

## Discussion

In this study, we established two nomograms for prognostic predictions for patients with stage I LUAD. Model 1 enabled the combination of the clinical T staging and IASLC grading system directly. Model 2 integrated clinical T staging with CT features which were predictive for IASLC histological grading. Comparing the prognostic performances of these two models, Model 2, which consisted mainly of preoperative CT image features, was slightly inferior to Model 1. While Model 1 was probably the best predictive model in our study, the clinical application value of Model 2 cannot be underestimated, as the predictors contained therein were readily available preoperatively. The Model 2 could provide us information about tumor prognosis before surgery, and then guide the preoperative adjuvant therapy for some patients at high risk of recurrence in the future. Moreover, for some patients who were unable to undergo or refused to receive surgery due to poor physical condition, advanced age or short-life expectancy et al, the Model 2 could provide evidence for developing appropriate management strategies. The time-dependent C-indexes of the two models were stable at approximately 0.9 and steadily superior to clinical T staging or histological grading alone (**Figure 4C**).

Previous studies have integrated T staging with histological subtypes to predict the prognosis of stage I lung cancer (4, 5, 11). However, most of these studies used the histological classification based on the predominant pattern or exploratory reclassification proposed by researchers (4, 5, 11). To our knowledge, few previous studies have combined the new IASLC grading system and T staging, especially clinical T staging, to predict the DFS of stage I lung adenocarcinoma patients. In our study, Model 1 achieved a combination of T staging and IASLC grading system and showed favorable prediction performance. On the other hand, several previous models were based on the combination of pre- and post-operative predictors (12, 13), which means that these models could only be used for patients who had already received surgery. However, some stage I LUAD patients are not eligible for surgery. Predicting tumor prognosis before treatment is crucial for treatment decisions for them. The predictive accuracy may be limited when using clinical T staging alone. Therefore, we identified additional preoperative predictors viable for prognostic stratification by assessing the relationship between radiological features and the new IASLC grading system and integrated them with clinical T staging in Model 2. Wang et al. found that the combination of eight TNM classifications and the pathological reclassification proposed by the authors was superior to TNM alone in predicting the early recurrence of stage I LUAD, but there was no marked difference in long-term recurrence and survival prediction (4). However, our results differed slightly from theirs; our two models had better prognostic discrimination than cT staging alone and performed stably over time. One possible reason is the superiority of the IASLC grading system, and the inconsistency between the cT and pT stages may also be one of the reasons (14).

Clinical T stage was the strongest of the preoperative predictors included in our models. Consistent with previous studies (15), our results validated the prognostic potential of the current cT categorization system, which is based on the solid portion size rather than the overall tumor size. In our study, cTis/cT1mi/cT1a, cT1b, cT1c, and cT2a effectively stratified patient outcomes. We found that pure GGNs classified as cTis were not necessarily pTis. Seventy-nine cases of invasive adenocarcinoma cancer (IAC) were pure GGN, of which 53.2% were histologic grade 1, 46.8% were grade 2, and none were grade 3. Sun et al. reported no recurrence in patients with a pure ground-glass mass of >3 cm, even for cases of IAC (16). Our study revealed that not only pure GGN but also LUADs with maximum solid component diameters of ≤1 cm on CT images had favorable prognoses. In our results, none of the 183 patients classified as cTis, cT1mi, and cT1a had recurrence after a median follow-up of 70.4 months, and patients with solid component sizes of >1 cm (≥cT1b) were at risk of recurrence, each 1-cm increase in the solid portion size increased the risk of recurrence by 2.32 times.

CTR is associated with the prognosis of LUAD (17). Xi et al. revealed that subsolid nodules could not be stratified effectively according to the CTR with an increment of 25% based on their prognostic role (18), which was consistent with our results. We found that CTR of <25% and ≥75% stratified patients with different prognoses. None of the 165 patients with tumor CTR of <25% had recurrence in our study. Yano et al. evaluated the prognosis of 1737 patients with clinical stage IA NSCLCs who underwent sublobar resection and reported that carcinomas with CTR of <25% rarely recur and were good candidates for limited resection (19). Some previous studies reported that patients with tumor size of ≤3 cm with CTR of ≤50% had very good prognoses (20, 21). In our study, three patients with CTR >25% and <50% had a relapse, accounting for 15% of relapsed patients with part-solid nodules and 5% of all relapsed patients. Our results revealed that CTR of ≥75% were associated with poor prognoses, and there was no significant difference between the Kaplan-Meier curves for part-solid nodules with CTRs of ≥75% and <100% and solid nodules with CTR of 100%. Kamigaichi et al. compared the cumulative incidence of recurrence between patients with nearly pure-solid (CTR ≥75%) and those with pure-solid tumors and found that for clinical T1c tumors, part-solid nodules with CTR ≥75% had similar cumulative incidence of recurrence as pure-solid nodules (*P=*0.130) (22). These results indicated that nearly pure-solid tumors may have similar prognoses as pure-solid tumors, and part-solid tumors with CTRs of ≥75% may be indicative of solid tumors for the choice of surgical modality and the development of postoperative management strategies.

Our results suggest that old age is significantly associated with worse prognoses, which has been demonstrated in several previous studies (23, 24). Xie et al. suggested that an age of 65 or more years is a risk factor for recurrence in patients with stage I lung adenocarcinoma (25). Regarding sex, several studies identified the male sex as a risk factor for poor prognosis (15, 23), however, sex was not an independent predictor in our study. We hypothesized that there was at least one independent predictor, such as CT features, in the final model that was significantly different for females and males and masked the risk of gender. We found that the proportion of tumors with CTR < 25%, histologic grade 1 was higher and with burr sign was less in females as compared to males, and female patients were significantly younger than male patients (**Table S** in the supplementary material). Therefore, the differences between men and women were represented by these predictors even though sex was not included in our final model.

The burrs sign has been verified to be an important indicator for differentiating peripheral lung cancer from inflammatory pseudotumor (26), and few studies have confirmed its prognostic value for lung cancer. Our findings may be partly influenced by the high proportion of recurrence of pure-solid nodules (mostly with burrs sign). Another noteworthy result is the multiple primary LUADs. Of the 379 patients in our study, 31.4% (119/379) had synchronous multifocal GGN(s), and these patients did not have significantly different DFS from those without them. We noticed that some previous prognostic studies excluded patients with multiple primary LUADs (4, 20), and our results suggest that cases presenting with multiple GGNs may be included.

This study has several limitations. First, it was a retrospective study, and analysis bias was inevitable. Second, owing to the low recurrence rate of stage I LUAD, the proportion of positive cases in our study was low (60/379), which may have affected our prognostic models. Third, the patients with stage cT2a were few (10/379), all of them had a relapse, and their recurrence rate may have been overestimated. Finally, this was a single-center study, and the prognostic values of the nomograms were not validated by external data. Future large-scale prospective multicenter studies are warranted to further validate our findings.

In conclusion, larger CTR and whole tumor size and higher mean CT value were independent predictors of higher histologic grade. CTR (cut-off values of <25% and ≥75%) and whole tumor size (cut-off value of 17 mm) could be used as preoperative surrogates for the IASLC grading system. The model based on CTR, whole tumor size, and other preoperative predictors, including clinical T stage, showed satisfactory performance in predicting the DFS of stage I LUAD patients.

## Data availability statement

The raw data supporting the conclusions of this article will be made available by the authors, without undue reservation.

## Ethics statement

The studies involving human participants were reviewed and approved by Institutional Ethics Committee of the Cancer Hospital, Chinese Academy of Medical Sciences. Written informed consent for participation was not required for this study in accordance with the national legislation and the institutional requirements.

## Author contributions

NW conceived, designed, and supervised the study. ML, WT, FT, HZ, CG, and FF acquired the data. ML and WT performed a visual analysis of the data. ML prepared the original draft. WT revised the paper. All authors qualify as per ICJME criteria for authorship. All authors contributed to the article and approved the submitted version.
