# Supplementary material for: Preoperative prognostic prediction for stage I lung adenocarcinomas: Impact of the computed tomography features associated with the new histological grading system
Source: Front Oncol. 2023 Jan 31;13:1103269. doi: 10.3389/fonc.2023.1103269 (PMC9927203; doi:10.3389/fonc.2023.1103269)
Supplement: Supplementary file 1 [file Table_1.docx]

**Supplementary material**

**Table S.** Differences between female and male in independent prognostic predictors

|  | female | male | statistic | *P* value |
| --- | --- | --- | --- | --- |
| age(y) | 57.86 | 59.76 | 2.27 | 0.024 |
| clinical T stage |  |  | 7.61 | 0.055 |
| cTis/cT1mi/cT1a | 121 | 62 |  |  |
| cT1b | 70 | 53 |  |  |
| cT1c | 35 | 28 |  |  |
| cT2a | 3 | 7 |  |  |
| burrs sign |  |  | 10.30 | 0.001 |
| yes | 81 | 78 |  |  |
| no | 148 | 72 |  |  |
| histologic grade |  |  | 8.52 | 0.014 |
| grade 1 | 37 | 20 |  |  |
| grade 2 | 150 | 83 |  |  |
| grade 3 | 42 | 47 |  |  |
| consolidation tumor ratio |  |  | 7.93 | 0.019 |
| <25% | 109 | 56 |  |  |
| ≥25% to <75% | 46 | 24 |  |  |
| ≥75% | 74 | 70 |  |  |
| whole tumor size |  |  | 3.30 | 0.069 |
| ≤17mm | 118 | 63 |  |  |
| >17mm | 111 | 87 |  |  |
